# Complete chloroplast genomes of three important species, *Abelmoschus moschatus*, *A*. *manihot* and *A*. *sagittifolius*: Genome structures, mutational hotspots, comparative and phylogenetic analysis in Malvaceae

**DOI:** 10.1371/journal.pone.0242591

**Published:** 2020-11-25

**Authors:** Jie Li, Guang-ying Ye, Hai-lin Liu, Zai-hua Wang

**Affiliations:** Guangdong Provincial Key Lab of Ornamental Plant Germplasm Innovation and Utilization, Environmental Horticulture Research Institute, Guangdong Academy of Agricultural Sciences, Guangzhou, China; National Cheng Kung University, TAIWAN

## Abstract

*Abelmoschus* is an economically and phylogenetically valuable genus in the family Malvaceae. Owing to coexistence of wild and cultivated form and interspecific hybridization, this genus is controversial in systematics and taxonomy and requires detailed investigation. Here, we present whole chloroplast genome sequences and annotation of three important species: *A*. *moschatus*, *A*. *manihot* and *A*. *sagittifolius*, and compared with *A*. *esculentus* published previously. These chloroplast genome sequences ranged from 163121 bp to 163453 bp in length and contained 132 genes with 87 protein-coding genes, 37 transfer RNA and 8 ribosomal RNA genes. Comparative analyses revealed that amino acid frequency and codon usage had similarity among four species, while the number of repeat sequences in *A*. *esculentus* were much lower than other three species. Six categories of simple sequence repeats (SSRs) were detected, but *A*. *moschatus* and *A*. *manihot* did not contain hexanucleotide SSRs. Single nucleotide polymorphisms (SNPs) of A/T, T/A and C/T were the largest number type, and the ratio of transition to transversion was from 0.37 to 0.55. *Abelmoschus* species showed relatively independent inverted-repeats (IR) boundary traits with different boundary genes compared with the other related Malvaceae species. The intergenic spacer regions had more polymorphic than protein-coding regions and intronic regions, and thirty mutational hotpots (≥200 bp) were identified in *Abelmoschus*, such as *start-psbA*, *atpB-rbcL*, *petD-exon2-rpoA*, *clpP-intron1* and *clpP-exon2*.These mutational hotpots could be used as polymorphic markers to resolve taxonomic discrepancies and biogeographical origin in genus *Abelmoschus*. Moreover, phylogenetic analysis of 33 Malvaceae species indicated that they were well divided into six subfamilies, and genus *Abelmoschus* was a well-supported clade within genus *Hibiscus*.

## Introduction

Family Malvaceae consists of 244 genera and over 4200 species, and most of them are widely distributed in tropics and temperate regions [1]. According to the diverse morphological characteristics, this family could be divided into nine subfamilies, including Sterculioideae, Tilioideae, Malvoideae, Helicteroideae, Grewioideae, Dombeyoideae, Byttnerioideae, Brownlowioideae and Bombacoideae [2]. *Abelmoschus* is one of important genera in subfamily Malvoideae of family Malvaceae. This genus was previously placed within *Hibiscus*, and subsequently isolated by taxonomists due to genetic differences [3]. As currently defined, genus *Abelmoschus* contains 11 species, 4 subspecies and 5 varieties [4], and displays a variable habit, from annual to perennial, herbs to shrubs, and is distributed in Asia, Australia and southwestern Africa [5]. Most members of this genus are economically important plants, and used in agriculture, food and medicines. *A*. *esculentus* (okra) and *A*. *caillei* are widely cultivated as vegetables due to their tender pods [6–8]. *A*. *manihot* is a popular green leafy vegetable and its flowers have been applied in clinical treatment of burns, chronic kidney disease and oral ulcers owing to the flavonoids [9, 10]. *A*. *moschatus*, as an aromatic plant, could be suitable for medical or food uses to improve insulin sensitivity [11]. *A*. *sagittifolius* also has a long history of medicinal usage, and cadinane sesquiterpenoid glucoside extracted from the stem tubers exhibited antitumor activity [12]. Moreover, antioxidant, antimicrobial, wound healing, anti-inflammatory and immunomodulatory activities were also found in *Abelmoschus* species [13–16]. Seed oil and levels of oleic acid have also been reported in *Abelmoschus* [3].

Due to coexistence of wild and cultivated form and interspecific hybridization, genus *Abelmoschus* is controversial in systematics and taxonomy, such as taxonomic position of some *Abelmoschus* species and the relationships between *Abelmoschus* species and part of *Hibiscus* species [8]. In terms of morphological and cytological features, highly variable root, flower and fruit characters of *Abelmoschus* have been used extensively in classification system [17, 18]. Patil et al. found seed coat sculpturing and seed trichomes could be used as the diagnostic characters for many morphologically closely related species of *Abelmoschus* [5]. Fluorochrome-binding pattern of nine *Abelmoschus* species showed polyploidy was an important factor in the chromosome number variation and evolution in this genus [4]. Some researchers also used molecular markers to analyze genetic relationships of *Abelmoschus*, but most studied focused on genetic diversity within *A*. *esculentus* and *A*. *manihot* [2, 7, 9, 18, 19], molecular markers were relatively lacking in other species. Thus, new molecular tools were necessary to study the accurate phylogeny in *Abelmoschus*.

Chloroplast is characteristic organelle in plant cells, and crucial in the photosynthesis and biosynthesis of pigments, amino acids, starch and fatty acids [20, 21]. The chloroplast genome generally has a circular structure with a pair of inverted-repeats (IR) regions (further called IRa and IRb), a large single copy (LSC) region and a small single copy (SSC) region. Due to the small size, conserved structure and gene content, it has been applied for resolving phylogenetics, evolution, taxonomic issues, population genetics and environmental adaptability [22]. Although chloroplast genome sequences of *Abelmoschus esculentus* has been deposited in GenBank (NC_035234.1) [23], there are no systematic, comprehensive and comparative studies of chloroplast genome in *Abelmoschus*.

In this study, three chloroplast genomes of *A*. *moschatu*s, *A*. *manihot* and *A*. *sagittifolius* were sequenced and compared with the chloroplast genomes of *A*. *esculentus* (NC_035234.1) and related species in Malvaceae. Apart from gene content and structure organization, comparative studies were conducted to identify mutational hotspots in *Abelmoschus*, and a phylogenetic tree of 33 species in family Malvaceae were constructed. These results will be useful in developing molecular markers for resolving taxonomic issues of *Abelmoschus*, and elucidating the evolutionary and phylogenetic relationships in the family of Malvaceae.

## Materials and methods

### Plant material, DNA isolation and sequencing

The fresh leaves of *A*. *moschatus*, *A*. *manihot* and *A*. *sagittifolius* were collected from the experimental field of Guangdong academy of agricultural sciences (Guangzhou, China). All samples were frozen in liquid nitrogen immediately and stored at −80°C. Total DNA was extracted by Plant DNA Isolation Kit (Tiangen, Beijing, China). Paired-end (PE) library was constructed according to protocol of Illumina manual (San Diego, CA, USA), and then it was run on an Illumina NovaSeq platform (Genepioneer Biotechnologies, Nanjing, China) with PE150 sequencing strategy and 350 bp insert size.

### Chloroplast genome assembly and annotation

Raw reads of three *Abelmoschus* species were filtered using the software NGSQCToolkit V2.3.3. In order to reduce the complexity of sequence assembly, filtered reads were compared with the chloroplast genome database built by Genepioneer Biotechnologies (Nanjing, China) using Bowtie2 V2.2.4, and sequences on the alignment was used as the chloroplast genome sequence of samples [24]. Seed sequence was obtained by software SPAdes v3.10.1, and contigs was acquired by kmer iterative extend seed. Then, the contigs were connected as scaffolds by SSPACE v 2.0, and gaps were filled using Gapfiller v2.1.1 until the complete chloroplast genome sequence was recovered. Finally, quality control was adopted to ensure the accuracy of assembly results with the reference genome of *A*. *esculentus* (NC_035234.1).

The coding sequences and ribosomal RNA (rRNA) were obtained using software BLAST V 2.2.25 and HMMER V3.1 b2 after compared with the chloroplast genome database in National Center of Biotechnology Information (NCBI). Aragorn V1.2.38 software was used for transfer RNA (tRNA) prediction, then tRNA annotation information of chloroplast genome was obtained. Chloroplast genome maps were made by OrganellarGenomeDRAW (OGDRAW).

### Relative Synonymous Codon Usage (RSCU) and RNA editing sites

RSCU analysis of *A*. *moschatus*, *A*. *manihot*, *A*. *sagittifolius* and *A*. *esculentus* (NC_035234.1) was determined using MEGA v7.0, and value of RSCU greater than one was considered to be a higher codon frequency. The putative RNA editing sites were analyzed by PREP-cp (http://prep.unl.edu/) with default parameters [25].

### Simple Sequence Repeats (SSRs) and repeat sequences

The comparison of SSRs within four *Abelmoschus* species were identified using MISA (MIcroSAtellite identification tool) v1.0 with 8 for mononucleotide repeats, 5 for di- and 3 each for tri-, tetra-, penta- and hexanucleotide repeats. Software vmatch v2.3.0 was used to identify forward (F), reverse (R), palindromic (P), and complementary (C) repeats with minimum repeats size ≥30 bp and sequence similarity of 90%.

### Genetic divergence, substitutions and insertion/deletions (Indels) analysis

MAFFT ((Multiple Alignments using Fast Fourier Transform) V7.427 was used to perform global alignment of protein-coding genes, intergenic spacer (IGS) regions, and intron regions of complete chloroplast genome among *A*. *moschatus*, *A*. *manihot*, *A*. *sagittifolius* and *A*. *esculentus* (NC_035234.1), and the value of genetic divergence (π) was calculated using DNAsp5. With the reference genome of *A*. *moschatus*, different types of single nucleotide polymorphisms (SNPs) and Indels were determined in *Abelmoschus* using MAFFT program.

### Analysis of non-synonymous (Ka)/synonymous (Ks), IR scope and collinearity

In order to analyze substitution rates of Ka/Ks, the protein-coding genes of *A*. *moschatus* (as reference) was compared with *A*. *manihot*, *A*. *sagittifolius*, *A*. *esculentus*, and three related species in Malvoideae: *Hibiscus rosa-sinensis* (NC_042239.1), *Althaea officinalis* (NC_034701.2) and *Gossypium hirsutum* (NC_007944.1). Protein-coding genes of all this species were aligned with *A*. *moschatus* and analysed by MAFFT V7.427, and the Ka/Ks value was calculated by the KaKs_calculator 2.0 [26].

The contraction and expansion of the IR boundaries among the above seven species in Malvoideae were visualized between the four regions of the chloroplast genome (LSC/IRb/SSC/IRa) by Geneious R8.1. Meanwhile, the analysis of chloroplast sequence homology and collinearity was performed by Mauve software.

### Phylogenetic analysis

Phylogenetic analysis was performed using the chloroplast genomes of *A*. *moschatus*, *A*. *manihot*, *A*. *sagittifolius* and *A*. *esculentus*, along with related 29 species within the same family of Malvaceae. Their accession numbers were listed in S1 Table. All chloroplast genome sequences were aligned through MAFFT V7.427, and Indels were removed by TrimAl (V1.4.rev15), then phylogenetic tree was constructed under maximum composite likelihood method (GTRGAMMA model and bootstrap = 1000) using RAxML v8.2.10.

## Results

### Characterization of chloroplast genomes in *Abelmoschus* species

**Fig 1:**
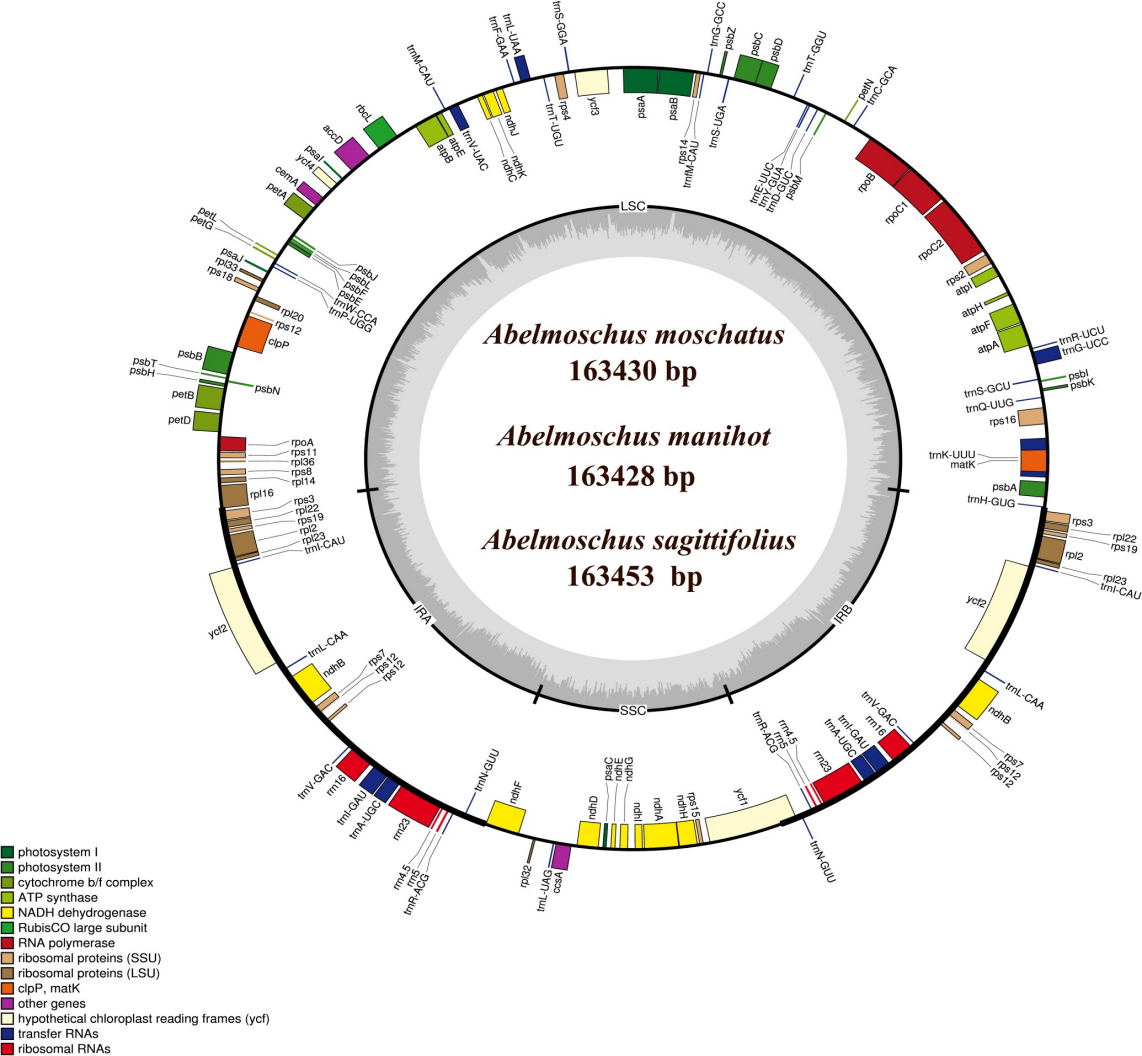
Chloroplast genome map of three *Abelmoschus* species. Genes shown outside the circle are transcribed clockwise and those inside counterclockwise. Genes belonging to different functional groups are color-coded.

Illumina Novaseq 6000 produced a total of 25,192,038, 19,864,607 and 21,300,029 paired-end (150bp) clean reads for *A*. *moschatus*, *A*. *manihot* and *A*. *sagittifolius*, with average organelle coverage 4470, 1888 and 3194, respectively. Chloroplast genome size was ~163 kb in *Abelmoschus* species, including a pair of IR regions separated by a LSC region and a SSC region (Fig 1 and Table 1). The GC content of *Abelmoschus* chloroplast genomes was ~36%, and the LSC, SSC and IR regions had similar content in four species, with ~34%, ~31% and ~41%, respectively.

**Table 1:** Summary statistics for the chloroplast genomes of *Abelmoschus* species.

Genome features	*A*. *moschatus*	*A*. *manihot*	*A*. *sagittifolius*	*A*. *esculentus*
Genome size (bp)	163430	163428	163453	163121
LSC size (bp)	88243	88194	88314	88071
SSC size (bp)	18931	18934	18815	19032
IR size (bp)	28128	28150	28162	28009
Number of genes	132(112)	132(112)	132(112)	131(111) ^a^
Protein genes [unique]	87(78)	87(78)	87(78)	87(78)
tRNA genes [unique]	37(30)	37(30)	37(30)	36(29) ^a^
rRNA genes [unique]	8(4)	8(4)	8(4)	8(4)
Duplicated genes in IR	20	20	20	20
GC content (%)	36.71	36.70	36.69	36.74
GC content in LSC (%)	34.47	34.48	34.45	34.55
GC content in SSC (%)	31.55	31.55	31.59	31.48
GC content in IR (%)	41.95	41.93	41.90	41.97

^a^ Data was from the *A*. *esculentus* chloroplast genome (NC_035234.1), and the number of genes should add one because gene *trnH-GUG* was not annotated.

The chloroplast genome of *Abelmoschus* species contained 132 genes (112 unique genes), including 87 protein-coding, 37 tRNA, and 8 rRNA genes (Table 2). Gene *trnH-GUG* was not annotated in the original annotation of *A*. *esculentus* (NC_035234.1). There are 20 duplicated genes, including four rRNA genes and 16 other genes (*ndhB*, *rpl2*, *rpl22*, *rpl23*, *rps12*, *rps19*, *rps3*, *rps7*, *trnA-UGC*, *trnI-CAU*, *trnI-GAU*, *trnL-CAA*, *trnN-GUU*, *trnR-ACG*, *trnV-GAC* and *ycf2*), and all of them repeats once. Moreover, 18 intron-containing genes were found (Table 3), fifteen of which contained one intron and three of which (*ycf3*, *trnV-UAC* and *clpP*) contained two introns. Except the genes of *trnA-UGC*, *trnI-GAU*, *ndhB*, *petD* and *petB*, thirteen other genes had different fragment sizes of intron. The complete chloroplast genome has been submitted to NCBI under GenBank accession numbers MT890968 for *A*. *moschatus*, MT898000 for *A*. *manihot*, and MT898001 for *A*. *sagittifolius*.

**Table 2:** List of annotated genes in the chloroplast genomes of *A*. *moschatus*, *A*. *manihot*, *A*. *sagittifolius* and *A*. *esculentus*.

Category	Gene group	Gene name
Photosynthesis	Subunits of photosystem I (5)	*psaA*, *psaB*, *psaC*, *psaI*, *psaJ*
	Subunits of photosystem II (15)	*psbA*, *psbB*, *psbC*, *psbD*, *psbE*, *psbF*, *psbH*, *psbI*, *psbJ*, *psbK*, *psbL*, *psbM*, *psbN*, *psbT*, *psbZ*
	Subunits of NADH dehydrogenase (12)	ndhA*, ndhB*(×2), *ndhC*, *ndhD*, *ndhE*, *ndhF*, *ndhG*, *ndhH*, *ndhI*, *ndhJ*, *ndhK*
	Subunits of cytochrome b/f complex (6)	*petA*, petB*, petD*, *petG*, *petL*, *petN*
	Subunits of ATP synthase (6)	*atpA*, *atpB*, *atpE*, atpF*, *atpH*, *atpI*
	Large subunit of rubisco (1)	*rbcL*
Self-replication	Proteins of large ribosomal subunit (12)	*rpl14*, rpl16*, rpl2*(×2), *rpl20*, *rpl22(×2)*, *rpl23(×2)*, *rpl32*, *rpl33*, *rpl36*
	Proteins of small ribosomal subunit (16)	*rps11*, rps12**(×2), *rps14*, *rps15*, rps16*, *rps18*, *rps19(×2)*, *rps2*, *rps3(×2)*, *rps4*, *rps7(×2)*, *rps8*
	Subunits of RNA polymerase (4)	*rpoA*, *rpoB*, rpoC1*, *rpoC2*
	Ribosomal RNAs (8)	*rrn16(×2)*, *rrn23(×2)*, *rrn4*.*5(×2)*, *rrn5(×2)*
	Transfer RNAs (37)	trnA-UGC*(×2), *trnC-GCA*, *trnD-GUC*, *trnE-UUC*, *trnF-GAA*, *trnG-GCC*, trnG-UCC*, *trnH-GUG*, *trnI-CAU(×2)*, trnI-GAU*(×2), trnK-UUU*, *trnL-CAA(×2)*, trnL-UAA*, *trnL-UAG*, *trnM-CAU*, *trnN-GUU(×2)*, *trnP-UGG*, *trnQ-UUG*, *trnR-ACG(×2)*, *trnR-UCU*, *trnS-GCU*, *trnS-GGA*, *trnS-UGA*, *trnT-GGU*, *trnT-UGU*, *trnV-GAC(×2)*, trnV-UAC*, *trnW-CCA*, *trnY-GUA*, *trnfM-CAU*
Other genes	Maturase (1)	*matK*
	Protease (1)	clpP**
	Envelope membrane protein (1)	*cemA*
	Acetyl-CoA carboxylase (1)	*accD*
	c-type cytochrome synthesis gene (1)	*ccsA*
unknown function	Conserved hypothetical chloroplast ORF (5)	*ycf1*, *ycf2(×2)*, ycf3**, *ycf4*

Gene*: Gene with one intron; Gene**: Gene with two introns; Gene (×2): Number of copies of multi-copy gene.

**Table 3:** Information on 18 intron-containing genes in the chloroplast genomes of *Abelmoschus* species.

Gene	Location	Exon I (bp)	Intron I (bp) ^a^	Exon II (bp)	Intron II (bp) ^a^	Exon III (bp)
*trnK-UUU*	LSC	37	2571/2563/2573/2576	35		
*rps16*	LSC	40	862/865/870/ 856	227		
*trnG-UCC*	LSC	23	803/809/804/ 811	48		
*atpF*	LSC	145	815/815/816/814	410		
*rpoC1*	LSC	432	773/774/781/780	1626		
*ycf3*	LSC	124	791/790/791/790	230	809/804/813/ 834	153
*trnL-UAA*	LSC	35	557/558/559/556	50		
*trnV-UAC*	LSC	38	590/590/590/608	35		
*rps12* ^*b*^	LSC/IRb	114	-	232	536/536/536/536	26
*clpP*	LSC	71	676 / 679/ 677/676	292	943/942/943/948	228
*petB*	LSC	6	812/812/812/821	642		
*petD*	LSC	8	757/757/757/757	475		
*rpl16*	IR	9	1148/1147/1147/ 1142	399		
*rpl2*	IR	391	698/698/698/696	434		
*ndhB*	IR	777	683/683/683/683	756		
*trnI-GAU*	IR	37	957/957/957/957	35		
*trnA-UGC*	IR	38	794/794/794/794	35		
*ndhA*	SSC	553	1119/1120/1119/1119	539		

^a^ The fragment size of intron is in the order of *A*. *moschatus / A*. *manihot / A*. *sagittifolius / A*.*esculentus*.

^b^ The *rps12* gene is divided into *5'-rps12* in the LSC region and *3'-rps12* in the IR region.

### Amino acid frequency, codon usage and RNA editing sites

Four *Abelmoschus* species showed similarity in amino acids frequency and codon usage. Protein-coding genes comprised 26713, 26705, 26714 and 26717 codons in *A*. *moschatus*, *A*. *manihot*, *A*. *sagittifolius* and *A*. *esculentus*, respectively (S2 Table and S1 Fig). Among those amino acids, Leucine was the most encoded amino acid followed by Isoleucine and Serine, while the Cysteine was the least abundant in chloroplast genomes. The use of the codons ATG and TGG, encoding Methionine and Tryptophan, exhibited no bias (RSCU = 1.00) in *Abelmoschus*. The findings also revealed that most of the amino acids preferred synonymous codons (RSCU >1.00) having A/T at 3′ end, except ATA and CTA encoding for Isoleucine and Leucine, respectively.

Putative RNA editing sites were also determined in four *Abelmoschus* species. PREP predicted 55 putative RNA editing sites in 24 genes of *A*. *moschatus* and *A*. *sagittifolius*, 56 putative RNA editing sites in 24 genes of *A*. *manihot*, and 62 putative RNA editing sites in 24 genes of *A*. *esculentus* (S3 Table). Similar RNA editing sites were found in most genes, however, gene *ycf3* was unique to *A*. *esculentus* and gene *clpP* was unique to *A*. *moschatus*, *A*. *manihot* and *A*. *sagittifolius*. The highest number of editing sites were determined in *ndhB* (12), *ndhD* (7), *matK*(5) and *petB* (5). Genes of *ndhD*, *ndhA* and *matK* varied widely variations among species: In *A*. *moschatus*, *A*. *manihot* and *A*. *sagittifolius* five and one RNA editing sites were found for *ndhD* and *ndhA*, while in *A*. *esculentus* seven and one RNA editing sites were present, respectively. *A*. *manihot* contained one more RNA editing sites in *matK* gene than other three species. Most conversion occurred at the first and second nucleotides of the codons, and mainly were C/G to A/T conversion. Change of RNA editing sites would produce abundant hydrophobic amino acids, especially Leucine, which was 29 in *A*. *moschatus*, *A*. *manihot* and *A*. *sagittifolius*, and 28 in *A*. *esculentus*.

### SSRs and repeat sequences

**Fig 2:**
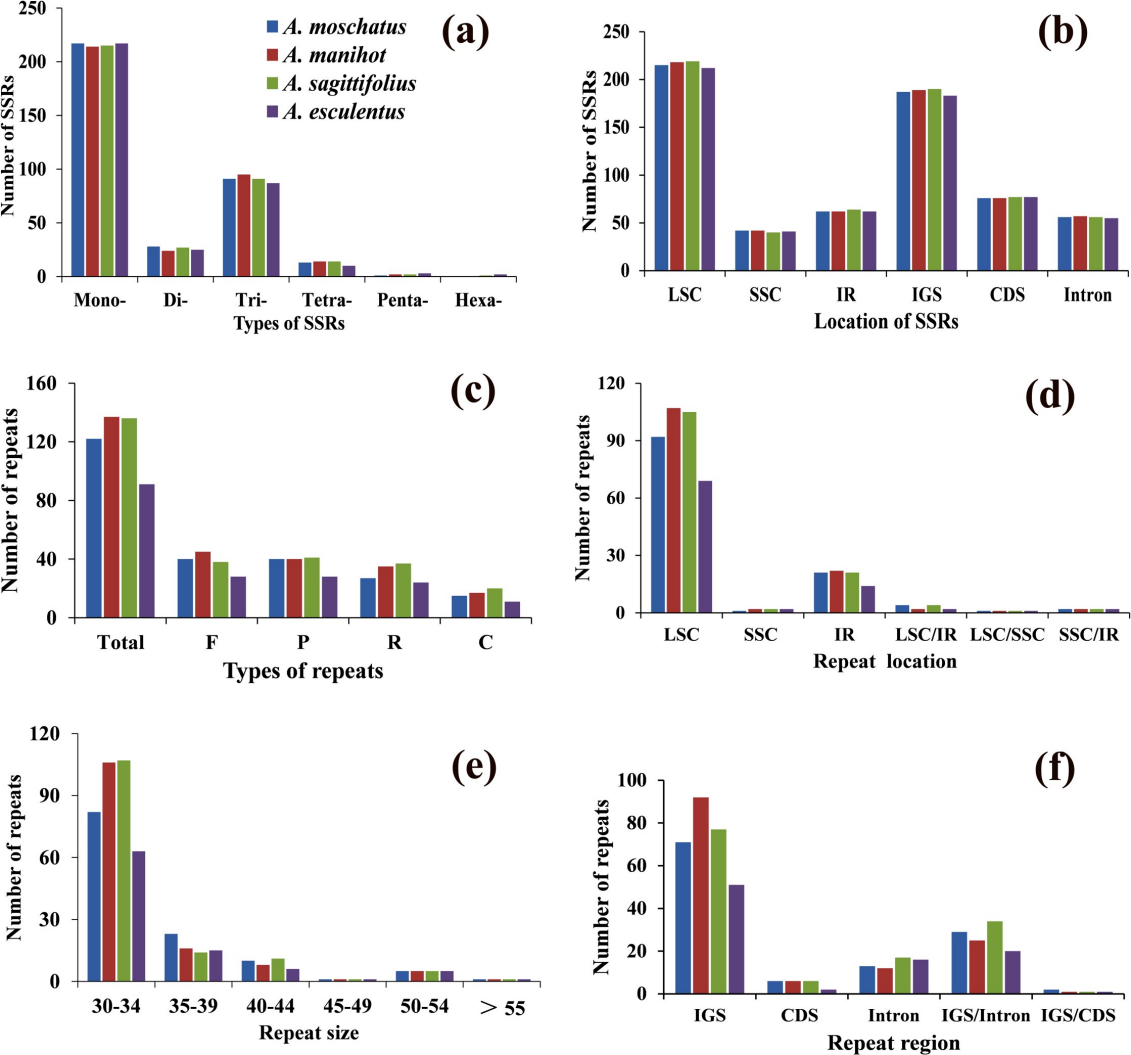
Comparison of SSRs and repeat sequences among four *Abelmoschus* species. (a) Numbers of different types of SSRs; Mono-: mononucleotide, Di-: dinucleotide, Tri-: trinucleotide, Tetra-: tetranucleotide, Penta-: pentanucleotide, Hexa-: hexanucleotide; (b) Location of SSRs in different chloroplast genome regions. LSC: large single copy, SSC: small single copy, IR: inverted-repeat region. IGS: Intergenic spacer regions, CDS: coding DNA sequences, Intron: intronic regions; (c) Different types of repeat sequences. Total: total numbers of all repeats. F: forward repeats, P: palindromic repeats, R: reverse repeats, C: complementary repeats; (d) Number of repeats present in different locations of chloroplast genomes. LSC/IR: one copy of repeat present in LSC and another in IR, LSC/SSC: one copy of repeat present in LSC and another in SSC, SSC/IR: one copy present in SSC and another in IR; (e) Number of repeats in different size. For example, 30–34 represent the numbers of repeats with the size from 30 to 34 bp; (f) Number of repeats in different regions of chloroplast genomes. IGS/Intron: one copy of repeat present in intergenic spacer regions and another in intronic regions. IGS/CDS: one copy of repeat present in intergenic spacer regions and another in coding regions.

SSRs were detected by MISA software in *Abelmoschus* (Fig 2A and 2B). *A*. *moschatus* contained 350, *A*. *manihot* (351), *A*. *sagittifolius* (350) and *A*. *esculentus* (344) SSRs. The maximum SSRs were mononucleotide and accounted for about 60% of total SSRs, varying in size from 8 to 18 nucleotides. Trinucleotide and dinucleotide SSRs were also abundant and accounted for about 33% of the total SSRs. *A*. *moschatus* and *A*. *manihot* did not contain hexanucleotides. The A/T and AT/TA were the most abundant mononucleotide and dinucleotide SSRs, respectively. The number of repeats units was also determined for all types of SSRs repeats (S4 Table). About 67% SSRs repeats were found in LSC, 13% in SSC, and 19% in IR. The IGS regions contained the most SSRs, and comprised approximately 58% of the total SSRs.

Four categories of repeat sequences were also found in *Abelmoschus*, and there were 486 repeats were present in the chloroplast genomes of four species, 122 in *A*. *moschatus*, 137 in *A*. *manihot*, 136 in *A*. *sagittifolius* and 91 in *A*. *esculentus* (Fig 2C–2F). Types of repeats (P, F and R) had similar numbers in each species, but the number of type C is relatively small. The size of repeats was mainly 30–54 bp in four *Abelmoschus*, and all contained one repeats above 55 bp. Abundant repeats were found in the IGS regions, followed by IGS/Intron regions. Meanwhile, most of the repeats were located in LSC (92, 107, 105, 69), followed by IR (21, 22, 21, 14) and lowest were in SSC (1, 2, 2, 2) in *A*. *moschatus*, *A*. *manihot*, *A*. *sagittifolius* and *A*. *esculentus*, respectively. We also found some shared sequences in LSC/SSC (all 1), SSC/IR (all 2), and LSC/IR (2–4) in four species. The complete details of repeat sequences in four *Abelmoschus* species were also listed in S5 Table.

### SNPs and Indels in *Abelmoschus*

**Fig 3:**
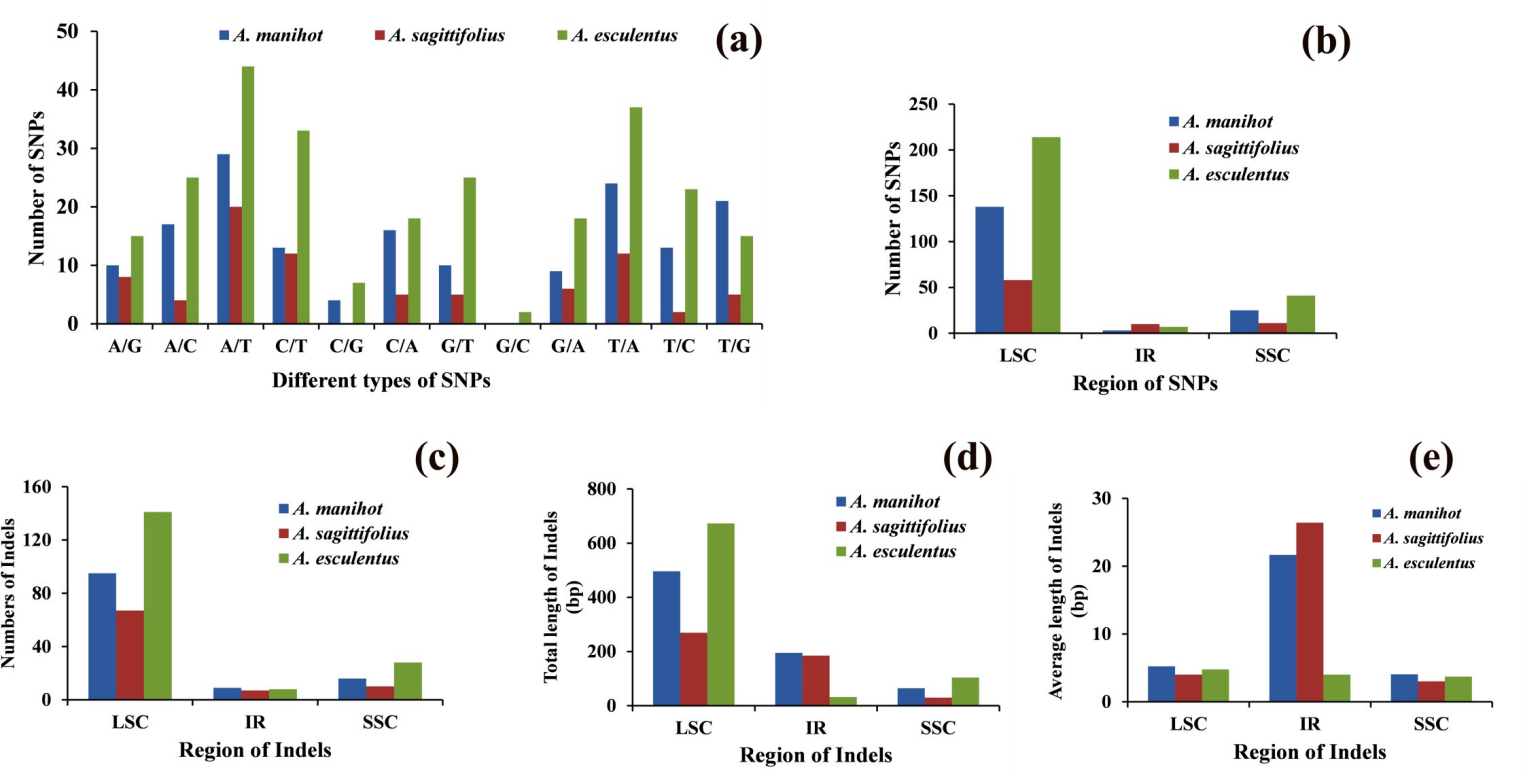
Comparison of SNPs and Indels in four *Abelmoschus* species. *A*. *moschatus* was used as reference for SNPs and Indels detection. (a) The number of different types of SNPs. (b) The number of SNPs in LSC, IR and SSC regions. (c) The number of Indels in LSC, IR and SSC regions. (d) Total length of Indels in LSC, IR and SSC regions. (e) Average length of Indels in LSC, IR and SSC regions. LSC: large single copy, SSC: small single copy, IR: inverted-repeat region.

Diverse types of SNPs were determined in four *Abelmoschus* species using *A*. *moschatus* as reference. *A*. *manihot*, *A*. *sagittifolius* and *A*. *esculentus* showed 166, 79 and 262 SNPs in complete chloroplast genome, respectively. SNPs of A/T, T/A and C/T were the largest number type among the 12 substitutions in *Abelmoschus* (Fig 3A and 3B), and most SNPs were located in LSC regions followed by SSC regions. The ratio of transition to transversion was 0.37 for *A*. *manihot*, 0.55 for *A*. *sagittifolius* and 0.51 for *A*. *esculentus*. Furthermore, Indels were also detected in different regions of chloroplast genomes. A total of 120, 84 and 177 Indels were found in *A*. *manihot*, *A*. *sagittifolius* and *A*. *esculentus*, and most of them existed in the LSC regions, but IR regions had the longest Indel average length in *A*. *manihot* and *A*. *sagittifolius* (Fig 3C and 3E).

### Mutational hotspots in *Abelmoschus*

**Fig 4:**
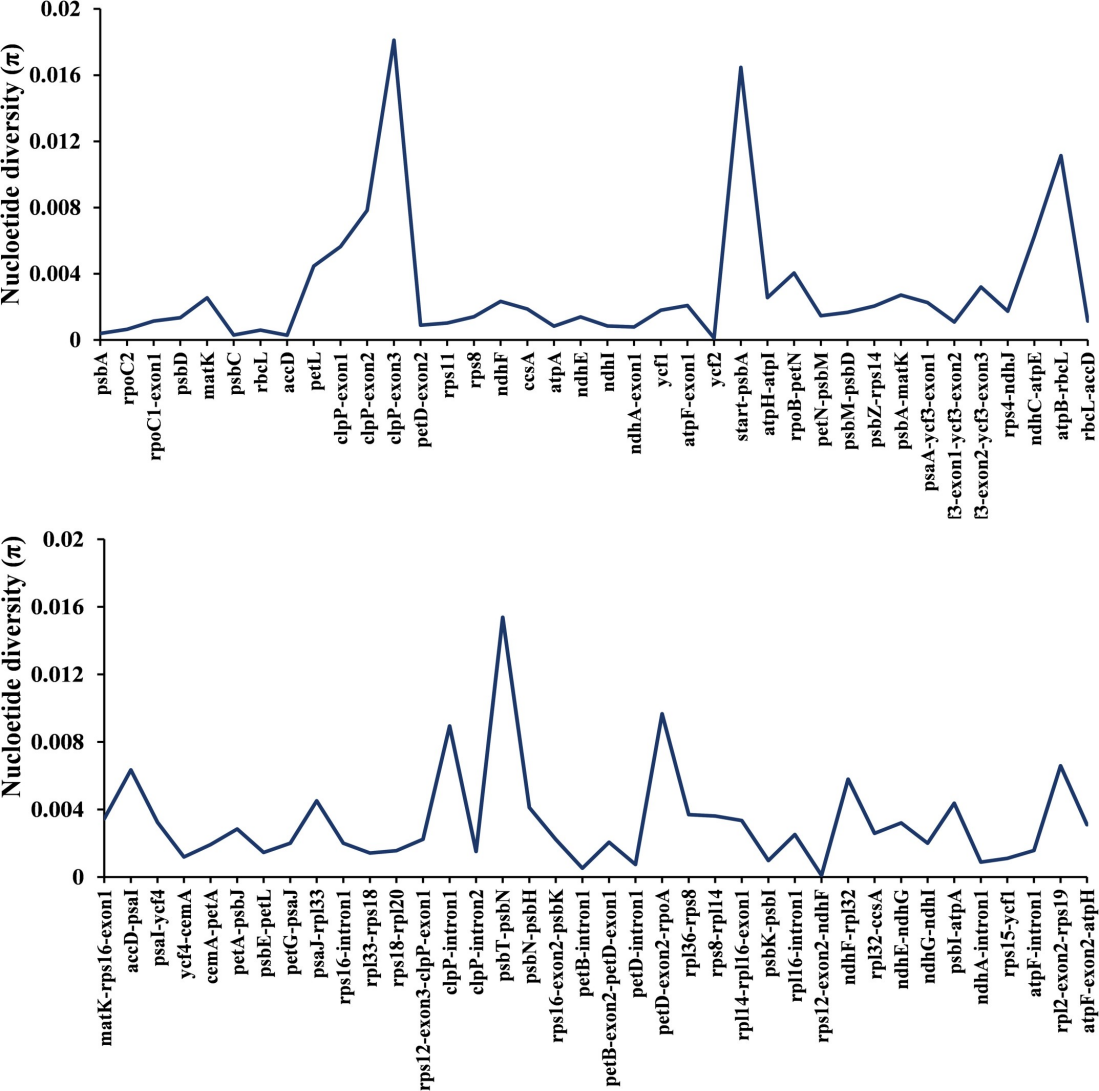
Nucleotide diversity (π) of different regions among *A*. *moschatus*, *A*. *manihot*, *A*. *sagittifolius* and *A*. *esculentus*. Regions with 0 nucleotide diversity were ignored. The x-axis represents chloroplast genome regions, and the y-axis represents nucleotide diversity.

Comparative analysis was conducted to identify mutational hotspots of protein-coding genes, IGS and intron regions of chloroplast genome among four *Abelmoschus* species. The IGS regions had more polymorphic (average π = 0.00432) compared to protein-coding regions (average π = 0.00285) and intronic regions (average π = 0.00269). The nucleotide diversity was ranged from 0.00013 (*rps12-exon2-ndhF*) to 0.02113 (*clpP-exon3*) in all the polymorphic containing regions (Fig 4). A total of thirty highly diverse regions (region length ≥200 bp) were listed in Table 4. Most of these mutational hotspots belong to IGS regions, such as *start-psbA*, *atpB-rbcL* and *petD-exon2-rpoA*. Higher nucleotide diversity was also observed for protein coding genes, including 1^st^ and 2^nd^ exon of *clpP*, 1^st^ intron of *clpP*, 2^nd^ intron of *ycf3*, *matK*, *ndhF* and 1^st^ exon of *atpF*.

**Table 4:** Mutational hotspots in four *Abelmoschus* species.

Region	Genetic divergence	Total Number of mutations	Region length
*start-psbA*	0.0165	23	642
*atpB-rbcL*	0.0111	30	1154
*petD-exon2-rpoA*	0.0097	6	266
*clpP-intron1*	0.0089	14	671
*clpP-exon2*	0.0078	5	292
*accD-psaI*	0.0063	11	743
*ndhC-atpE*	0.0063	33	2343
*ndhF-rpl32*	0.0058	11	862
*clpP-exon1*	0.0056	3	228
*psaJ-rpl33*	0.0045	5	475
*psbI-atpA*	0.0044	24	2380
*rpoB-petN*	0.0040	16	1907
*rpl36-rps8*	0.0037	4	464
*matK-rps16-exon1*	0.0035	13	1653
*psaI-ycf4*	0.0032	3	396
*ndhE-ndhG*	0.0032	2	267
*ycf3-intron2*	0.0032	6	802
*atpF-exon2-atpH*	0.0031	4	597
*petA-psbJ*	0.0028	6	1155
*psbA-matK*	0.0027	4	631
*rpl32-ccsA*	0.0026	7	1267
*atpH-atpI*	0.0026	7	1169
*matK*	0.0025	9	1515
*rpl16-intron1*	0.0025	6	1132
*ndhF*	0.0023	12	2196
*psaA-ycf3-exon1*	0.0023	5	948
*rps16-exon2-psbK*	0.0022	5	1023
*atpF-exon1*	0.0021	2	410
*petB-exon2-petD-exon1*	0.0021	1	207
*psbZ-rps14*	0.0021	5	1043

### IR boundary and collinearity

**Fig 5:**
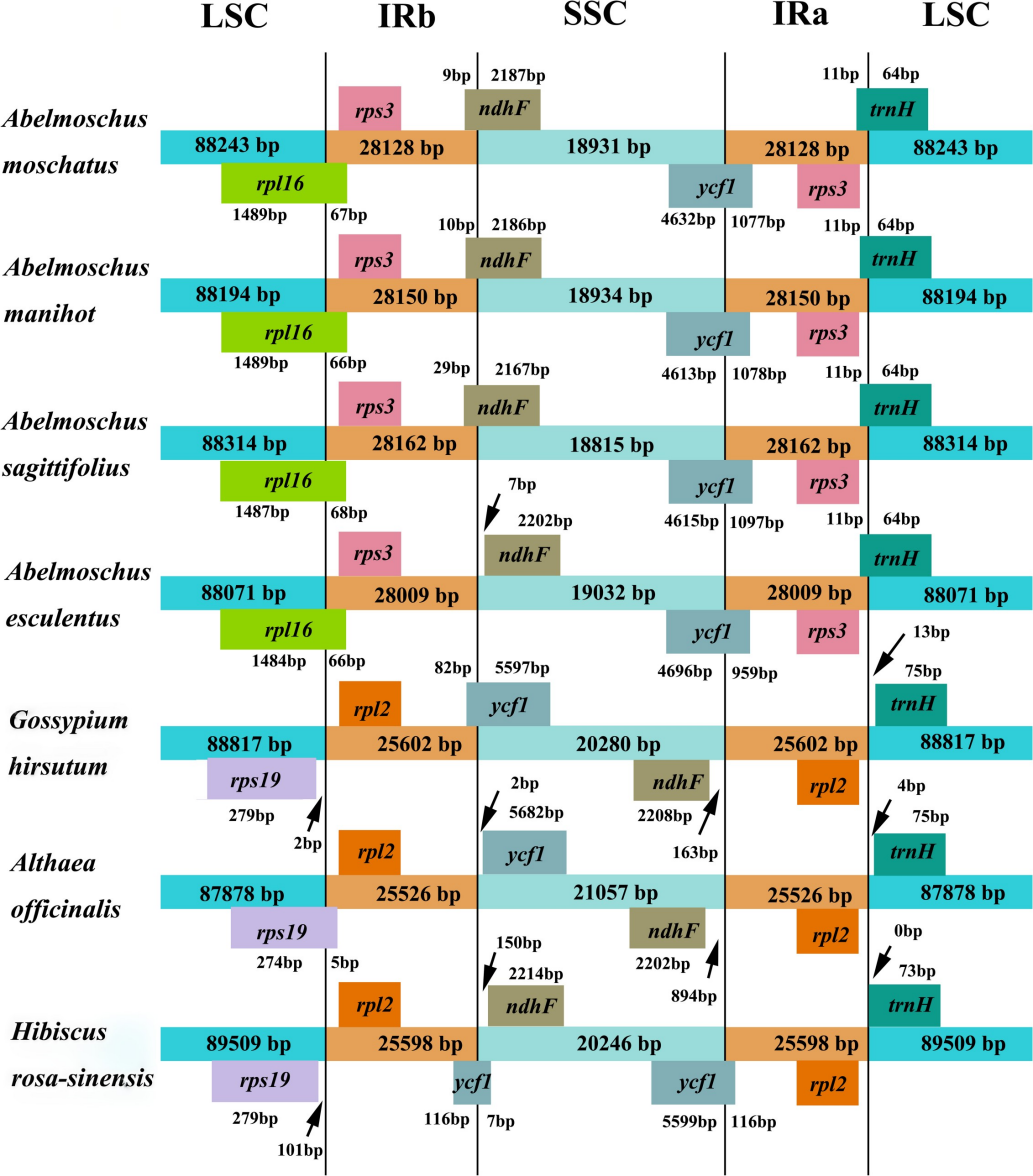
Comparative analysis of boundary regions: IR, SSC and LSC among four *Abelmoschus* species and three related species in Malvaceae.

**Fig 6:**
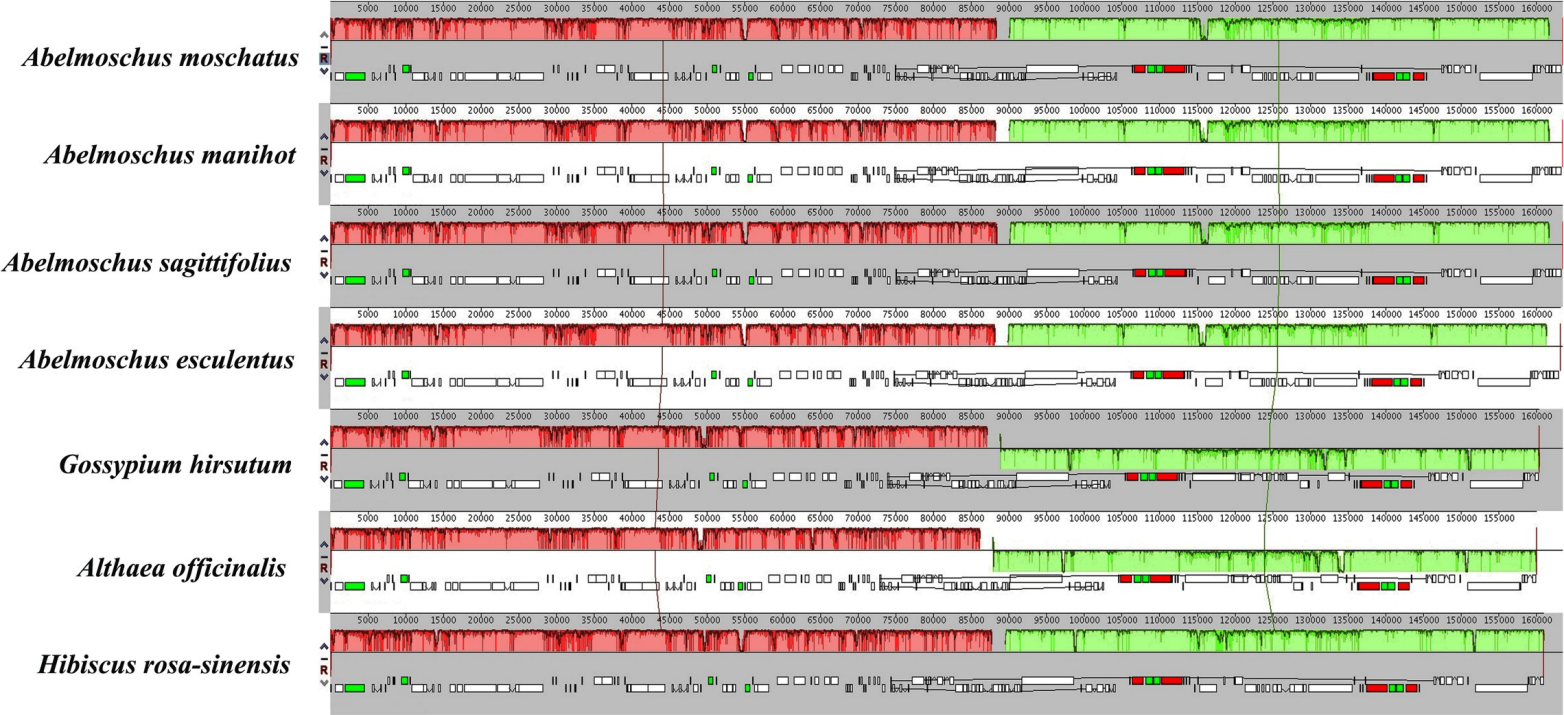
Co-linear analysis of seven Malvaceae chloroplast genomes. The *Abelmoschus moschatu*s genome is shown at top as the reference. Within each of the alignment, local collinear blocks are represented by blocks of the same color connected by lines.

The IR regions were compared among four *Abelmoschus* species and three closely related species in family Malvaceae (Fig 5). The *trnH* gene of *A*. *esculentus* was reannotated in the junction of IRa/LSC. In four *Abelmoschus* species, the LSC/IRb boundary was located within the coding region of *rpl16* gene, with 66 to 68 bp in the IRb region. The *ycf1* gene spanned the boundary of the SSC/IRa region, with 959–1097 bp in the IRa region. The IRb/SSC and IRa/LSC boundaries were crossed by the *ndhF* gene and *trnH* gene. However, *ndhF* gene was all located in SSC region, and 7 bp from the boundary in *A*. *esculentus*. The *trnH* gene had the same fragment size of 64 bp in LSC region of four *Abelmoschus*. Moreover, the genes of *rpl16*, *ndhF* and *ycf1* showed different fragment sizes of 1550-1556bp, 2196-2202bp and 5655-5712bp in four *Abelmoschus* species, respectively. Based on LSC/IRb/SSC/IRa boundaries, the relationships among *A*. *moschatus*, *A*. *manihot* and *A*. *sagittifolius* were closer than *A*. *esculentus*. In addition, the pseudogene fragment of *ycf1* was 123 bp in the junction of SSC/IRb in *Hibiscus rosa-sinensis*. The *trnH* gene was all located in LSC region, with 0-13bp from the boundary in *H*. *rosa-sinensis*, *Althaea officinalis* and *G*. *hirsutum*. The *rps19* gene was in junction of LSC/IRb in *A*. *officinalis*. The chloroplast genomes of 7 species were relatively conserved after aligned by Mauve software, and no rearrangement occurred in gene organization (Fig 6), but the gene layouts within SSC regions of *A*. *officinalis* and *G*. *hirsutum* were in the opposite orientations compared with *H*. *rosa-sinensis* and four *Abelmoschus* species.

### Ka/Ks substitution rate

In this study, we analyzed Ka/Ks rate of *A*. *moschatus* compared with to three species in the same genus and three closely related species in family Malvaceae (S6 Table). Eighty-five protein-coding genes were analyzed and thirty-eight of them had an average Ka/Ks rate between 0 to 0.1 in seven species, which indicated these genes were under strong purifying selection pressure in family Malvaceae. In contrast, three genes showed Ka/Ks>1.0, included gene *rpl23* in *H*. *rosa-sinensis* (1.12), *A*. *officinalis* (2.80) and *G*. *hirsutum* (1.79), gene *clpP* in *A*. *esculentus* (6.01) and *H*. *rosa-sinensis* (3.67), gene *ycf1* in *A*. *manihot* (1.50) and *A*. *esculentus* (2.98). In addition, gene *matK* had Ka/Ks = 1.0 in *A*. *esculentus*, and seven genes (*ndhA*, *ccsA*, *psbT*, *rps15*, *rbcL*, *accD* and *ycf2*) had Ka/Ks rate between 0.5 and 1.0 in at least one species.

### Phylogenetic analysis

**Fig 7:**
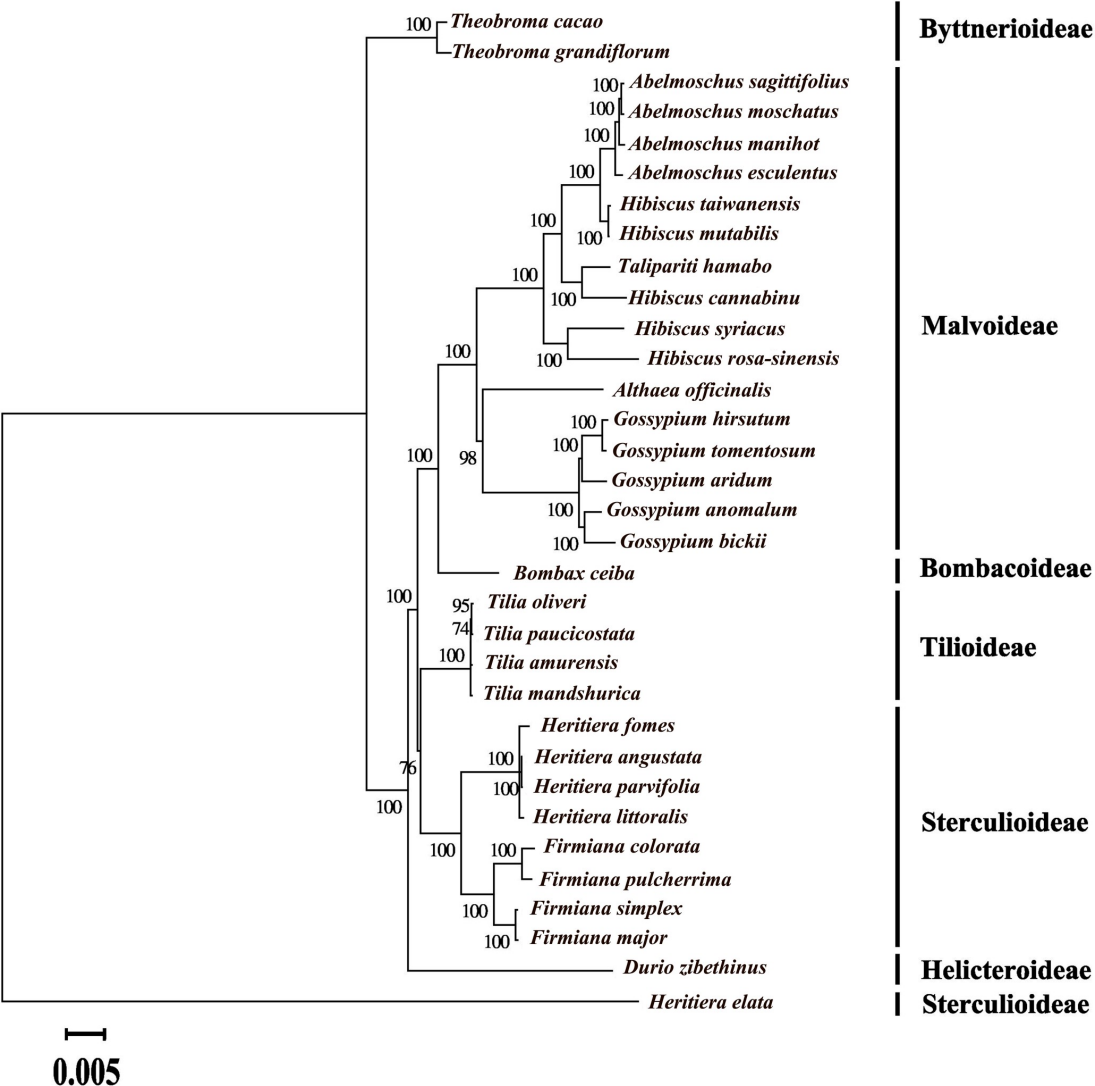
Maximum likelihood phylogenetics tree of 33 species in family Malvaceae based on chloroplast genomes (Indels removed).

Maximum likelihood phylogenetic tree of 33 species in family Malvaceae were constructed based on complete chloroplast genomes after removing the Indels. Phylogenetic analysis indicated that *A*. *moschatus* sister to *A*. *sagittifolius*, four *Abelmoschus* species shared a common node with *H*. *taiwanensis* and *H*. *mutabilis*, and then they came together with other *Hibiscus* species to form a large group. The species of six different subfamilies were well distinguished with bootstrap values about 100. However, Sterculioideae subfamily was divided into two groups because *Heritiera elata* did not share a same node with other species in the same genus (Fig 7).

## Discussion

### Genome characteristics of *Abelmoschus* species and comparison with other species in Malvaceae

Most species in *Abelmoschus* were economically important plants, but the chloroplast genomes remained relatively limited, with only *A*. *esculentus* was sequenced [23]. In this study, three chloroplast genomes of *A*. *moschatus*, *A*. *manihot* and *A*. *sagittifolius* were sequenced and compares with *A*. *esculentus*. The sizes of chloroplast genomes ranged narrowly from 163121 to 163453 in four *Abelmoschus* species, and comparative analyses revealed highly conserved structure and gene. Most angiosperms typically contained 74 to 79 protein-coding genes in chloroplast genomes [27]. In this study, four *Abelmoschus* species all encoded 78 unique protein-coding genes, and was different with previously reported species of *Hibiscus cannabinus* and three *Firmiana* speies in Malvaceae, which contain 79 protein-coding genes [22, 28]. Rabah et al. [23] reported *A*. *esculentus* had 29 unique tRNA genes, but the gene *trnH-GUG*, located at LSC/IRa boundary, was not annotated, so we reannotated this gene for this species. Within the same subfamily, previous studies reported 17, 19 and 18 intron-containing genes in *H*. *cannabinus*, *H*. *rosa-sinensis* and 12 species of *Gossypium*, respectively [2, 28, 29], while *Abelmoschus* harbored 18 intron-containing genes, thirteen out of them had intron length differences among 4 species, and gene *trnK-UUU* had the longest intron with 2563–2576 bp.

The chloroplast genomes had well collinearity relationship among four *Abelmoschus* species and three closely related species in Malvaceae, but some differences were detected in terms of the direction of SSC, gene miss and IR expansion and contraction. Gene layouts within SSC region had the same orientations between four *Abelmoschus* species and *H*. *rosa-sinensis*, but *A*. *officinalis* and *G*. *hirsutum* had the opposite orientations compared with them, and similar phenomenon with different inversions in the LSC region was also found in *Chenopodium quinoa* and *Mangifera indica* [23]. The *infA* gene as a translation initiation factor has been independently lost many times during the evolution of land plants [27, 30], and it also missed in *Abelmoschus*, but *infA* showed functional or non-functional in different Malvaceae species, such as *H*. *rosa-sinensis* [2].

The border of IR was highly variable region with many nucleotide changes in chloroplast genomes of closely related species. Among four *Abelmoschus* species, the genes of *rpl16*, *ndhF* and *ycf1* showed different fragment sizes in the IR boundaries, and the IRb/SSC border was crossed by the *ndhF* except *A*. *esculentus*, in which *ndhF* had larger gene size and all located in SSC region, this indicated that the relationships among *A*.*moschatus*, *A*. *manihot* and *A*. *sagittifolius* were closer than *A*. *esculentus*. Moreover, *Abelmoschus* species showed relatively independent boundary traits compared with the other Malvaceae species. Gene *rpl16* was located at the junction of IRb/LSC in *Abelmoschus*, whereas *A*. *officinalis* and *G*. *hirsutum* presented *rps19* gene crossing the boundary or locating in LSC region, and ten species in different genus of Malvaceae also showed *rps19* gene in IRb/LSC [2]. In the IRb region, *rps3* was the closest gene to the IRb/LSC boundary in *Abelmoschus*, but this gene was replaced by the *rpl2* in other Malvaceae species. *Durio zibethinus* was a Malvaceae species with another boundary characteristic, *rpl23* (in LSC) and *trnI-CAU* (in IRb) were the closest genes to the IRb/LSC boundary, and *rpl23* and *rpl2* had only one copy due to IR expansion and contraction [31]. These results seem to be line with phylogenetic analysis, which indicated that species with more similar boundary traits had closer phylogenetic relationship in Malvaceae.

### SSRs and repeat sequences in *Abelmoschus*

Owing to the advantages of non-recombination, haploidy, uniparental inheritance and low nucleotide substitution rate, chloroplast SSRs markers can be considered as an excellent tool in population genetics and phylogeny analysis [32]. In the current study, mononucleotide SSR in four *Abelmoschus* species varied in size from 8 to 18 nucleotides, which was different from related species in *Hibiscus* (7 to 15 nucleotides) and *Firmiana* (7 to 22 nucleotides) [2, 22]. Both of *A*. *sagittifolius* and *A*. *esculentus* had six types SSRs, but *A*. *moschatus* and *A*. *manihot* did not contain hexanucleotides. Most SSRs were distributed in LSC region and intergenic region, and the identified SSRs in *Abelmoschus* revealed that A/T and AT/TA were the most abundant in mononucleotide and dinucleotide SSRs respectively, which agreed with the majority of plant family [24]. Moreover, repeat sequences was lower in *A*. *esculentus* compared with *A*. *moschatus*, *A*. *manihot* and *A*. *sagittifolius*, but they shared similar distribution regions. Abundant repeats were found in the intergenic spacer regions (IGS), followed by intronic region and coding sequences, and the same distribution pattern of repeat sequences were also reported in *Hibiscus* [2]. These repeat sequences were also crucial in chloroplast genome arrangement and sequence variation of *Abelmoschus*.

### Taxonomic discrepancies and hotspots in *Abelmoschus*

Previous studies reported that seed shape and trichome structure had major taxonomic importance and proved to be valuable characters for separating taxa of *Abelmoschus* [5]. SSR markers (mainly in *A*. *esculentus*) were also developed from transcriptome data and genomic DNA to investigate genetic relatedness and cross-species transferability [7, 19]. Pfeil et al. analyzed the phylogeny of *Hibiscus* and the Tribe Hibisceae using chloroplast DNA sequences of *ndhF* and *rp116* intron, and found two tested *Abelmoschus* species were embedded within *Hibiscus* [33]. Werner et al. [8] used nuclear internal transcribed spacer (ITS) and chloroplast *rpl16* sequences to construct phylogenetic relationships within *Abelmoschus*, and its relationship with the genus *Hibiscus* and other related species in Malvaceae, but *A*. *esculentus* and *A*. *caillei* cannot be distinguished from each other, and genetic diversity within *A*. *esculentus* and *A*. *caillei* was low. In this study, we listed thirty highly mutational hotspots (≥200 bp) after comparing nucleotide diversity of protein-coding genes, IGS, and intron regions among *Abelmoschus*, and these hotspots could be used to solve taxonomic discrepancies for genus *Abelmoschus*. Most mutational hotspots belong to IGS regions, and some hotspots in protein-coding genes had also been commonly used for barcoding markers in related genera, such as *matK*, *rbcL* and *ndhF* [2, 33]. The nucleotide diversity of *rpl16* intron was 0.0029, while the thirty hotspots identified in *Abelmoschus* had nucleotide diversity from 0.0024 to 0.0142, and 23 regions had higher polymorphic than previously reported sequence of *rpl16*. Interestingly, three exons and one intron of *clpP* gene all showed high nucleotide diversity, especially the third exon (π = 0.02113, region length = 71bp), and polymorphic region of this gene had been proved to be effective in evaluating the crop types and biogeographical origin of *Cannabis sativa* [34]. Therefore, all these mutational hotspots provided useful information for subsequent development of chloroplast markers, evolutionary relationships and biogeographical origin.

### Phylogenetic relationship in Malvaceae

Phylogenetic tree of 33 species in family Malvaceae were reconstructed using chloroplast genomes without Indels in this study, and they were well divided into six subfamilies, except *Heritiera elata* which did not share a node with other species in the same genus. Few previous studies referred to the taxonomic position of *A*. *sagittifolius*, our phylogenetic tree suggested that *A*. *sagittifolius* was closer to *A*. *moschatus* than to *A*. *esculentus*, which was consistent with the morphological characteristics of pod and flower [12]. Furthermore, genus *Abelmoschus* was previously included in the genus *Hibiscus* and later isolated from it [3]. Four *Abelmoschus* species shared a common node with *H*. *taiwanensis* and *H*. *mutabilis*, and then they formed a large group with other *Hibiscus* species located in different branches. These results indicated that *Abelmoschus* was a well-supported clade within *Hibiscus*, and agreed with the viewpoint of Werner et al. [8]. Thus, taxonomic treatment of *Abelmoschus* is an issue that required further discussion. *Abelmoschus* could be merged with *Hibiscus* to form a broad genus *Hibiscus*, or it maintains the taxonomic position of *Abelmoschus*, but some *Hibiscus* species need to change their taxonomic position. As more complete chloroplast genomes are sequenced, the chloroplast genome data could be expected to help resolve the deeper branches of phylogeny and complex evolutionary histories in Malvaceae [35].

## Conclusions

Three chloroplast genomes of *A*. *moschatu*s, *A*. *manihot* and *A*. *sagittifolius* were sequenced and annotated in the present study, and compared with the chloroplast genomes of *A*. *esculentus* and related species in Malvaceae. The results revealed the gene number and order, amino acid frequency, and codon usage were similar in *Abelmoschus*. However, the differences were also found in IR boundaries, intron-containing genes and the number of repeat sequences and SNPs. *Abelmoschus* species also showed relatively independent IR boundary traits compared with related species in Malvaceae, and identified thirty mutational hotpots might be useful for developing molecular markers and resolving taxonomic discrepancies and biogeographical origin both at genus *Abelmoschus* and family Malvaceae levels.

## Supporting information

S1 Fig Amino acids frequency in *A*. *moschatus*, *A*. *manihot*, *A*. *sagittifolius* and *A*. *esculentus*. (TIF)

S1 Table Accessions of 33 species used in phylogenetic tree. (DOCX)

S2 Table Comparison of Relative Synonymous Codon Usage (RSCU) among *A*. *moschatus*, *A*. *manihot*, *A*. *sagittifolius* and *A*. *esculentus*. (DOCX)

S3 Table RNA editing sites in *A*. *moschatus*, *A*. *manihot*, *A*. *sagittifolius* and *A*. *esculentus*. (XLSX)

S4 Table Details of SSRs in *A*. *moschatus*, *A*. *manihot*, *A*. *sagittifolius* and *A*. *esculentus*. (XLSX)

S5 Table Details of repeat sequences in *A*. *moschatus*, *A*. *manihot*, *A*. *sagittifolius* and *A*. *esculentus*. (XLSX)

S6 Table Rate of synonymous and non-synonymous substitutions. *Abelmoschus moschatus* (as reference genome) was compared with *A*. *manihot*, *A*. *sagittifolius*, *A*. *esculentus*, and three closely related species in Malvoideae: *Hibiscus rosa-sinensis* (NC_042239.1), *Althaea officinalis* (NC_034701.2) and *Gossypium hirsutum* (NC_007944.1). Eighty-five protein-coding genes were analyzed.
(XLSX)
