# Supplementary figures and images for: Complete chloroplast genomes of three important species, Abelmoschus moschatus, A. manihot and A. sagittifolius: Genome structures, mutational hotspots, comparative and phylogenetic analysis in Malvaceae
Source: PLoS One. 2020 Nov 25;15(11):e0242591. doi: 10.1371/journal.pone.0242591 (PMC7688171; doi:10.1371/journal.pone.0242591)

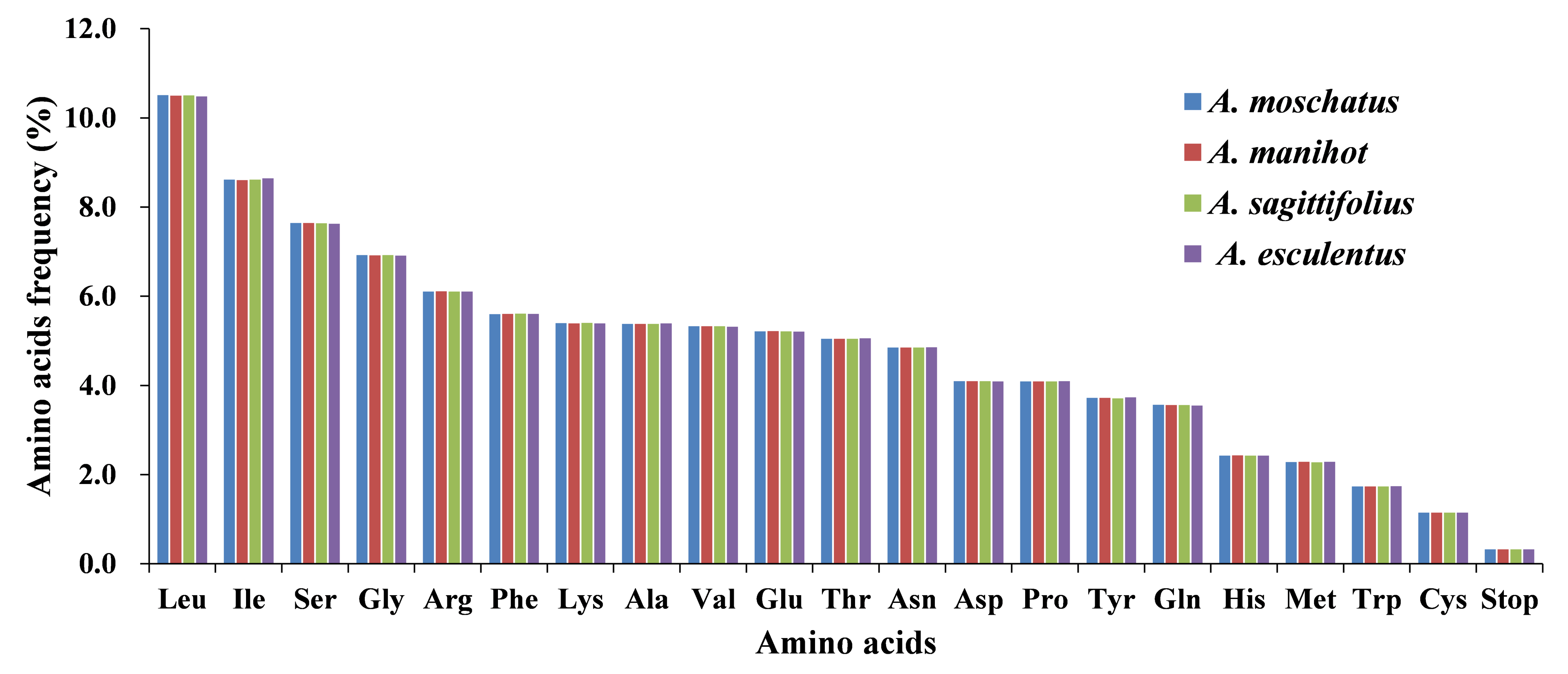

Supplement: S1 Fig — (TIF) [file pone.0242591.s001.tif]
