# Supplementary material for: Complete chloroplast genomes of three important species, Abelmoschus moschatus, A. manihot and A. sagittifolius: Genome structures, mutational hotspots, comparative and phylogenetic analysis in Malvaceae
Source: PLoS One. 2020 Nov 25;15(11):e0242591. doi: 10.1371/journal.pone.0242591 (PMC7688171; doi:10.1371/journal.pone.0242591)
Supplement: S1 Table — (DOCX) [file pone.0242591.s002.docx]

**S1 Table. Accessions of 33 species used in phylogenetic tree.**

| No. | Species | Accession numbers |
| --- | --- | --- |
| 1 | *Abelmoschus moschatus* | MT890968 |
| 2 | *Abelmoschus manihot* | MT898000 |
| 3 | *Abelmoschus sagittifolius* | MT898001 |
| 4 | *Abelmoschus esculentus* | NC_035234.1 |
| 5 | [*Talipariti hamabo*](https://www.ncbi.nlm.nih.gov/nuccore/NC_030195.1) | NC_030195.1 |
| 6 | [*Hibiscus cannabinus*](https://www.ncbi.nlm.nih.gov/nuccore/NC_045873.1) | NC_045873.1 |
| 7 | [*Hibiscus taiwanensis*](https://www.ncbi.nlm.nih.gov/nuccore/MK937807.1) | MK937807.1 |
| 8 | [*Hibiscus rosa-sinensis*](https://www.ncbi.nlm.nih.gov/nuccore/NC_042239.1) | NC_042239.1 |
| 9 | [*Hibiscus syriacus*](https://www.ncbi.nlm.nih.gov/nuccore/NC_026909.1) | MH330684.1 |
| 10 | *Hibiscus mutabilis* | MK820657.1 |
| 11 | [*Althaea officinalis*](https://www.ncbi.nlm.nih.gov/nuccore/NC_034701.1) | NC_034701.1 |
| 12 | *Bombax ceiba* | NC_037494.1 |
| 13 | *Durio zibethinus* | NC_036829.1 |
| 14 | *Theobroma cacao* | HQ336404.2 |
| 15 | *Theobroma grandiflorum* | JQ228388 |
| 16 | *Firmiana pulcherrima* | NC_036395.1 |
| 17 | *Firmiana simplex* | NC_041438.1 |
| 18 | *Firmiana major* | NC_037242.1 |
| 19 | *Firmiana colorata* | BK010724.1 |
| 20 | *Heritiera littoralis* | NC_043923.1 |
| 21 | *Heritiera angustata* | NC_037784.1 |
| 22 | *Heritiera elata* | NC_043925.1 |
| 23 | *Heritiera fomes* | NC_043924.1 |
| 24 | *Heritiera parvifolia* | NC_038057.1 |
| 25 | *Tilia amurensis* | MH169579.1 |
| 26 | *Tilia paucicostata* | NC_028591.1 |
| 27 | *Tilia oliveri* | NC_028590.1 |
| 28 | *Tilia mandshurica* | NC_028589.1 |
| 29 | *Gossypium hirsutum* | NC_007944.1 |
| 30 | *Gossypium bickii* | NC_023214.1 |
| 31 | *Gossypium anomalum* | NC_023213.1 |
| 32 | *Gossypium tomentosum* | NC_016690.1 |
| 33 | *Gossypium aridum* | NC_033396.1 |
