# Supplementary material for: Complete chloroplast genomes of three important species, Abelmoschus moschatus, A. manihot and A. sagittifolius: Genome structures, mutational hotspots, comparative and phylogenetic analysis in Malvaceae
Source: PLoS One. 2020 Nov 25;15(11):e0242591. doi: 10.1371/journal.pone.0242591 (PMC7688171; doi:10.1371/journal.pone.0242591)
Supplement: S2 Table — (DOCX) [file pone.0242591.s003.docx]

**S2 Table. Comparison of relative synonymous codon usage (RSCU) among *A. moschatus, A. manihot, A. sagittifolius* and *A. esculentus*.**

| Codon | Amino Acid | *A. moschatus* | *A. manihot* | *A. sagittifolius* | *A. esculentus* |
| --- | --- | --- | --- | --- | --- |
| TAA | * | 1.759 | 1.759 | 1.759 | 1.724 |
| TAG | * | 0.621 | 0.621 | 0.621 | 0.655 |
| TGA | * | 0.621 | 0.621 | 0.621 | 0.621 |
| GCA | A | 1.065 | 1.066 | 1.066 | 1.070 |
| GCC | A | 0.662 | 0.662 | 0.662 | 0.661 |
| GCG | A | 0.512 | 0.515 | 0.515 | 0.514 |
| GCT | A | 1.761 | 1.756 | 1.756 | 1.756 |
| TGC | C | 0.508 | 0.510 | 0.508 | 0.508 |
| TGT | C | 1.492 | 1.490 | 1.492 | 1.492 |
| GAC | D | 0.399 | 0.399 | 0.399 | 0.399 |
| GAT | D | 1.601 | 1.601 | 1.601 | 1.601 |
| GAA | E | 1.482 | 1.482 | 1.483 | 1.480 |
| GAG | E | 0.518 | 0.518 | 0.517 | 0.520 |
| TTC | F | 0.705 | 0.705 | 0.704 | 0.703 |
| TTT | F | 1.295 | 1.295 | 1.296 | 1.297 |
| GGA | G | 1.577 | 1.576 | 1.577 | 1.581 |
| GGC | G | 0.420 | 0.422 | 0.420 | 0.414 |
| GGG | G | 0.727 | 0.730 | 0.727 | 0.728 |
| GGT | G | 1.276 | 1.273 | 1.276 | 1.278 |
| CAC | H | 0.503 | 0.505 | 0.503 | 0.503 |
| CAT | H | 1.497 | 1.495 | 1.497 | 1.497 |
| ATA | I | 0.938 | 0.939 | 0.938 | 0.938 |
| ATC | I | 0.592 | 0.591 | 0.593 | 0.594 |
| ATT | I | 1.471 | 1.470 | 1.469 | 1.468 |
| AAA | K | 1.490 | 1.490 | 1.490 | 1.489 |
| AAG | K | 0.510 | 0.510 | 0.510 | 0.511 |
| CTA | L | 0.857 | 0.858 | 0.857 | 0.859 |
| CTC | L | 0.408 | 0.407 | 0.408 | 0.401 |
| CTG | L | 0.394 | 0.394 | 0.394 | 0.392 |
| CTT | L | 1.250 | 1.250 | 1.250 | 1.249 |
| TTA | L | 1.851 | 1.851 | 1.849 | 1.868 |
| TTG | L | 1.240 | 1.241 | 1.242 | 1.232 |
| ATG | M | 1.000 | 1.000 | 1.000 | 1.000 |
| AAC | N | 0.477 | 0.475 | 0.477 | 0.473 |
| AAT | N | 1.523 | 1.525 | 1.523 | 1.527 |
| CCA | P | 1.128 | 1.131 | 1.128 | 1.122 |
| CCC | P | 0.751 | 0.750 | 0.751 | 0.746 |
| CCG | P | 0.553 | 0.553 | 0.553 | 0.552 |
| CCT | P | 1.568 | 1.566 | 1.568 | 1.580 |
| CAA | Q | 1.532 | 1.533 | 1.532 | 1.530 |
| CAG | Q | 0.468 | 0.467 | 0.468 | 0.470 |
| AGA | R | 1.777 | 1.772 | 1.777 | 1.784 |
| AGG | R | 0.692 | 0.691 | 0.692 | 0.692 |
| CGA | R | 1.387 | 1.390 | 1.387 | 1.383 |
| CGC | R | 0.471 | 0.470 | 0.471 | 0.471 |
| CGG | R | 0.394 | 0.394 | 0.394 | 0.394 |
| CGT | R | 1.280 | 1.283 | 1.280 | 1.277 |
| AGC | S | 0.362 | 0.362 | 0.362 | 0.359 |
| AGT | S | 1.156 | 1.158 | 1.156 | 1.163 |
| TCA | S | 1.270 | 1.267 | 1.271 | 1.267 |
| TCC | S | 0.964 | 0.958 | 0.965 | 0.955 |
| TCG | S | 0.538 | 0.535 | 0.532 | 0.545 |
| TCT | S | 1.711 | 1.720 | 1.715 | 1.711 |
| ACA | T | 1.205 | 1.206 | 1.202 | 1.208 |
| ACC | T | 0.766 | 0.766 | 0.766 | 0.767 |
| ACG | T | 0.492 | 0.493 | 0.496 | 0.494 |
| ACT | T | 1.537 | 1.535 | 1.537 | 1.531 |
| GTA | V | 1.493 | 1.496 | 1.493 | 1.495 |
| GTC | V | 0.509 | 0.509 | 0.509 | 0.507 |
| GTG | V | 0.559 | 0.559 | 0.559 | 0.560 |
| GTT | V | 1.439 | 1.436 | 1.439 | 1.438 |
| TGG | W | 1.000 | 1.000 | 1.000 | 1.000 |
| TAC | Y | 0.406 | 0.408 | 0.403 | 0.403 |
| TAT | Y | 1.594 | 1.592 | 1.597 | 1.597 |
